# Porcine Tuberculosis

**Published:** 1906-02-24

**Authors:** 


### The Hospital.

A Journal of

The Medical Sciences and Hospital Administration.

Vol. XXXIX.—No. 1,013. SATURDAY, FEBRUARY 24, 1906.

### Porcine Tuberculosis.

An interesting report issued by the Medical Department of the Local Government Board contains a timely reminder that the diffusion of tuberculosis may be occasioned in many other ways than by the agency of human sputa furnished by phthisical patients. It has long been known, not only that the pig is subject to tuberculosis, but also that the disease, when affecting animals of this species, is apt to become much more rapidly generalised or diffused over their bodies than is the case with bovine animals, in whom it may remain localised in an udder or other organ for a comparatively long period of time. It was also established, by the Royal Commission on Tuberculosis of 1898, that bacilli situate in the deeper portions of a joint of meat, or in the inner layers of a butcher's "roll," were neither killed nor deprived of their infective activity by ordinary cooking. In these circumstances Dr. G. S. Buchanan was instructed to discover what precautions are actually taken to secure the quality of pig's meat, and to what extent these precautions may be relied upon as sources of safety to the consumer. It cannot be said that his report is in the highest degree encouraging.

With regard to the actual amount of prevalence of tuberculosis in the pig, it has been found difficult or impossible to arrive at any exact conclusions. There are certain abattoirs, both in the United Kingdom and abroad, in which it seems to be carefully looked for, and in which the percentages discovered are comparatively small; but there is much reason to believe that these abattoirs are not attractive to the owners of pigs likely to be diseased. The percentages actually discovered range from 1.0 or 1.5 to nearly 6.0; and even the smaller of these, when considered in relation to the enormous numbers of animals dealt with, must be regarded as representing a very large supply of diseased and dangerous meat. The Government of the Netherlands has for some time past been taking active steps for the promotion and encouragement of an extensive export trade in pork, more especially to ourselves, and for this purpose has established a vigilant system of inspection in all the abattoirs from which the carcases of pigs are sent out of the country; and similar care seems to be taken in some portions of the German Empire, but not in others. In the United States, at least so far as the great factories are concerned, the vast quantities dealt with appear to place formidable difficulties in the way of any really effective inspection; and, as a matter of course, nothing of the kind can even be expected with regard to what may be described as the domestic slaughtering of individual farmers, either in Great Britain or in Ireland. At the Central Meat Market in the City there is a general inspection, by competent officials, of the carcases consigned to salesmen, and by them offered to retail butchers; but the system under which it is conducted affords many possibilities of evasion. The salesmen themselves are accustomed to call the attention of the inspectors to any consignment which appears to them to be of a suspicious character; but, failing this, the chances that any given carcase will be examined seem to be somewhat remote.

The pig is said to become infected with tuberculosis mainly through its food, and especially from the proximity of tubercular cows, and from obtaining portions of their milk. It follows that the first signs of tuberculosis in the pig are usually to be found in the sub-maxillary and cervical glands, or in the abdominal glands, and hence it is important that these should be readily accessible to observation. Dr. Buchanan tells an instructive story of a pork dealer and sausage maker on a large scale on whose premises diseased meat was discovered by an inspector, and who ever since has had every carcase reaching him stripped of glands and connective tissue with the least possible delay, and these possibly tell-tale portions of his merchandise consigned with all speed to the mincing machine. As far as the flesh and fat are concerned, it is obvious that they might be in a high degree tubercular without betraying the fact to any ordinary form of examination. Dr. Buchanan is quite clear as to the advisability of a more strict examination of porcine carcases than is at present possible, and is strongly in favour of Sir Shirley Murphy's proposal of reception stations for all dead meat entering the metropolis, stations at which it would be properly inspected, and from which it would issue guaranteed by the official stamp of the inspector. Until something like this is done it should not be forgotten by the housewife that pig's meat, in all its forms, is subject to a condition the dangers from which may be obviated by thorough cooking, and that the very general distaste for "underdone" pork is based upon a healthy instinct which should not be neglected.

